# The role of mesenchymal stem cells in chemotherapy-induced gonadotoxicity

**DOI:** 10.1186/s13287-018-0946-6

**Published:** 2018-07-18

**Authors:** Iman O. Sherif, Dina Sabry, Azza Abdel-Aziz, Osama M. Sarhan

**Affiliations:** 1 Emergency Hospital, Faculty of Medicine, Mansoura University, Mansoura, 35516 Egypt; 2 Medical Biochemistry and Molecular Biology Department, Faculty of Medicine, Cairo University, Cairo, 11562 Egypt; 3 Pathology Department, Faculty of Medicine, Mansoura University, Mansoura, 35516 Egypt; 4 Urology and Nephrology Center, Faculty of Medicine, Mansoura University, Mansoura, 35516 Egypt

**Keywords:** Cisplatin, Mesenchymal stem cells, Oxidative stress, Inflammation, Apoptosis, Testicular toxicity

## Abstract

**Background:**

The therapeutic potential of bone marrow-derived mesenchymal stem cells (BM-MSCs) against cisplatin-induced nephrotoxicity has been reported, however, its efficacy in gonadotoxicity still has not been addressed. Herein, we investigated the effect of BM-MSCs in cisplatin-induced testicular toxicity and its underlying mechanism of action.

**Methods:**

Thirty male Sprague–Dawley rats were divided into a control group: injected with phosphate-buffered saline (PBS) intraperitoneal (ip), a cisplatin group: injected with a single dose of 7 mg/kg cisplatin ip to induce gonadotoxicity and a BM-MSCs group: received cisplatin ip followed by BM-MSCs injection 1 day after cisplatin. In testicular tissues, malondialdehyde (MDA), superoxide dismutase (SOD), and reduced glutathione (GSH) levels were assessed. Additionally, gene expressions of inducible nitric oxide synthase (iNOS), caspase-3, and p38 mitogen-activated protein kinase (MAPK) were measured. The testicular tumor necrosis factor alpha (TNF-α) protein contents and Bcl-2 associated X protein (BAX) expression were determined. Histopathology of testicular tissues was examined.

**Results:**

Cisplatin injection showed a significant decrease in GSH and SOD testicular levels besides a significant increase of MDA and TNF-α testicular levels and upregulation of testicular gene expressions of iNOS, caspase-3, and p38-MAPK in comparison to the control group. Moreover, a marked increase in BAX protein expression was observed in the cisplatin group when compared with the control one. Histopathological examination exhibited significant seminiferous tubules atrophy in cisplatin-treated rats.

**Conclusions:**

The BM-MSCs injection significantly repaired the testicular injury and improved both biochemical and histopathological changes. The MSCs mitigated the gonadotoxicity induced by cisplatin through antioxidative, anti-inflammatory, and antiapoptotic mechanisms.

## Background

Cisplatin a chemotherapeutic drug is used in the treatment of various solid tumors [1]. Despite high efficiency, its application is associated with significant side effects including toxicity in many organs such as kidney, liver, and testis [2–4]. In animal models, cisplatin caused reproductive toxicity including germ cell apoptosis, azoospermia, and testicular atrophy [2–5]. The gonadotoxicity mechanism induced by cisplatin is not fully yet understood; however, several studies have shown that cisplatin exposure can lead to oxidant/antioxidant imbalance, which elicits oxidative damage of DNA, proteins, and lipids [2, 3, 6]. Anti-oxidant defense systems such as catalase, superoxide dismutase (SOD) and reduced glutathione (GSH), are involved in protecting tissues from the reactive oxygen species (ROS) damage [7].

Recently, stem cell biology has been focused on the antioxidative role of stem cells and its application to repair tissues from ROS damaging effects [7, 8]. Mesenchymal stem cells (MSCs) are kind of multipotent stem cells isolated from many tissues like bone marrow, adipose tissue, cord blood, and amniotic membrane [9]. They are characterized by specific surface antigen expression in addition to osteogenic, chondrogenic, and adipogenic differentiation; they also have the ability to self-renew [7, 10, 11]. It was documented that bone marrow-derived mesenchymal stem cells (BM-MSCs) could repair the testicular tissue damage that occurred in busulfan-treated infertile mice and hamsters [12, 13], lead [7], and cadmium-treated rats [14, 15]. Transplanted BM-MSCs could be localized to the basal membrane of the seminiferous tubule, where they improved the testicular tissue architecture [12, 16]. This improvement effect of MSCs was attributed to the secretion of growth factors and cytokines by stem cells leading to immunomodulatory and antiapoptotic activities [2, 14, 16, 17].

Successful use of MSCs in several animal models of different organ toxicity [2, 7, 14, 17–19] lead us to conduct the current study to evaluate for the first time the effect of BM-MSCs against testicular damage induced by cisplatin and investigate its molecular mechanism.

## Methods

### Animals

Adult male Sprague–Dawley rats with an average weight of 200–250 g were kept under standard conditions of illumination with a 12 h light/dark cycles at (25° ± 2 °C) and allowed free access to food and tap water.

### Isolation and culture of BM-MSCs

The BM cells were isolated as described before by Elawady et al., 2016 [20]. In brief, BM cells were flushed from tibia and fibula of rat bones with phosphate-buffered saline (PBS). Over 15 ml Ficoll-Paque (Gibco-Invitrogen, Grand Island, NY, USA), flushed BM cells were layered, centrifuged, and the upper layer was discarded leaving a layer of a mononuclear cell at the interphase, which was then collected, washed twice in PBS, and centrifuged. Isolated BM-MSCs were cultured, propagated, and supplemented with 10% fetal bovine serum (FBS), 0.5% penicillin/streptomycin and incubated at 37 °C and 5% CO_2_ until reaching 80–90% confluence within 7 days.

### Characterization and transplantation of BM-MSCs

The MSCs were identified by their morphology, adherence, surface markers, and their capacity to differentiate into osteocytes and adipocytes as detected by an inverted microscope (Leica, Wetzlar, Germany). Cell surface antigen expression of BM-MSCs was analyzed by flow cytometry in which cells were incubated with fluorescein isothiocyanate-conjugated (anti-CD45) or phycoerythrin- cyanine-5-conjugated (anti-CD90) or rat immunoglobulin G (IgG) isotype control antibodies for 30 min at 4 °C in PBS [20].

Furthermore, the adipocytes differentiation was achieved by adipocytes StemPro® adipogenesis differentiation kit (Gibco, Life Technology, Carlsbad, CA, USA) and they were stained by Oil Red O stain (Sigma-Aldrich, St Louis, MO, USA). The osteocytes differentiation was achieved by osteocytes StemPro® osteogenesis differentiation kit (Gibco, Life Technology) and they were stained by Alizarin Red S stain (Sigma- Aldrich).

### Experimental design

Thirty male rats were randomly assigned to three experimental groups (group = 10) as follows: normal control group: injected intraperitoneally (ip) with PBS, cisplatin group: injected with cisplatin dissolved in PBS (7 mg/kg, ip) (Hospira, Leamington Spa, UK) to induce testicular toxicity [3, 21] , and the BM-MSCs group: injected with cisplatin (7 mg/kg, ip) and on the next day 2 × 10^6^ BM-MSCs dissolved in PBS by intravenous injection [17]. This 1-day interval was designed mimicking previous study protocols that have been performed using BM-MSCs in cisplatin-induced nephrotoxicity rat models [17, 22, 23].

### Sample collection

After 7 days, animals were euthanized and blood samples were collected by cardiac puncture into heparinized tubes for testosterone evaluation. Testicular tissues were dissected and removed from the scrota and then immediately weighed. A part of the testis was homogenized in PBS. The homogenates were stored at − 80 °C until used for further biochemical investigations. For real-time PCR assay, 30 mg of testis was preserved in liquid nitrogen. Testicular tissues for histopathological examination were immersed-fixed in Bouin’s solution for 2 days.

### Determination of testosterone levels

The plasma testosterone levels were measured as described in previous studies using testosterone ELISA kits (Abcam, Inc., Cambridge, UK) for rats according to the manufacturer’s instructions [3, 24, 25]. The assay is based on competitive ELISA where testosterone in the sample competes with the added testosterone-horseradish peroxidase (HRP) for antibody binding. In brief, a 96-well plate was pre-coated with anti-testosterone antibodies. Samples and the testosterone-HRP conjugate were added to the wells, Next, the wells were washed and 3,3',5,5'-Ttetramethylbenzidine (TMB) substrate was added to produce blue coloration. Stop Solution was added to stop the color development and produce yellow color. The intensity of the signal was inversely proportional to the content of testosterone in the sample which was measured at 450 nm.

### Assay of oxidative stress levels

Testicular levels of malondialdehyde (MDA), reduced glutathione (GSH) and superoxide dismutase (SOD) were measured using kits from Biodiagnostic Co., Giza, Egypt.

### Measurement of tumor necrosis factor alpha (TNF-α)

Testicular levels of TNF-α was estimated as described in previous studies using rat TNF-α ELISA kit (USCN Life Science, Houston, TX, USA) [17, 26] based on a sandwich enzyme immunoassay. The manufacturer’s instructions were followed. Standards or samples were added to the microtiter plate wells with a biotin-conjugated antibody specific to TNF-α. Afterwards, avidin conjugated to HRP was added and incubated. After the addition of TMB substrate solution, wells that contained TNF-α, biotin-conjugated antibody and enzyme-conjugated avidin would display a change in color. The enzyme-substrate reaction was ended by the addition of sulfuric acid and the color change was determined at 450 nm.

### Real time-polymerase chain reactions (PCR)

Testicular gene expressions of inducible nitric oxide synthase (iNOS), mitogen-activated protein kinase (MAPK) and caspase-3 were assessed by real time-PCR quantitative analysis. Total RNA was isolated from testicular tissue homogenates using RNeasy Purification Reagent (Qiagen, Valencia, CA, USA). RNA concentration was determined by spectrophotometer and the RNA integrity was studied by gel electrophoresis on a 1% agarose gel. The cDNA was produced from 2 μg of total RNA samples using Oligo(dT)12–18 and SuperScript Reverse Transcriptase (Life Technologies, Breda, the Netherlands). The primers for real-time PCR were: iNOS forward primer: 5′-AAGAGTTCCCATCATTGCGT-3′ and reverse primer: 5′-TC CTCAACCTGCTCCTCACT-3′; p38-MAPK forward primer:5′-ACTCAGATGCCGAAGATGAAC-3′ and reverse primer: 5′- GTGCTCAGGACTCCATCTCT-3′; caspase-3 forward primer:′5-TGTCATCTCGCTCTGGTACG-3′ and reverse primer: 5′-AAATGAC-CCCTTCATCACCA-3′ and beta-actin forward primer: 5′-TCT GGC ACC ACA CCT TCT- ACA ATG-3′ and reverse primer: 5′- AGC ACA GCC TGG ATA GCA ACG-3′. Real-time quantitative PCR was done by using SYBR Green PCR Master Mix (Applied Biosystems, Foster City, CA, USA). PCR reactions were done in step one plus Real-Time PCR system (Applied Biosystems). Relative expressions of the studied genes were estimated by the comparative threshold cycle method. Normalization of values to beta-actin genes was performed [17].

### Histopathology examination

Histopathological examination of 4-μm- thick testicular sections stained with hematoxylin and eosin (H&E) was performed on an Olympus CX31 light microscope (Olympus, Tokyo, Japan). Pictures were obtained by a PC-driven digital camera (Olympus E-620). The diameters of the seminiferous tubules were measured. Fifty random tubular cross-sections per testicular section were examined at ×400 magnification, totaling 100 seminiferous tubule sections per animal [27].

### Immunohistochemistry evaluation

Immunohistochemistry of Bcl-2 associated X protein (Bax) expression was evaluated by using polyclonal anti-Bax antibody (Novus Biologicals, Littleton, CO, USA) at dilutions of 1:1000 and measured by the streptavidin–biotin complex method. The selected paraffin blocks for immunohistochemical staining was sectioned and pre-treated by boiling in 10 mM Tris buffer containing 1 mM EDTA (pH 6.0) for 30 min then incubated with the primary antibody for 70 min at room temperature [14].

### Statistical analysis

Data were expressed as mean ± SD. To detect the differences between groups one-way ANOVA followed by Bonferroni multiple tests was applied. Significance was considered when *p* ≤ 0.05. Statistical analyses were performed using SPSS version 20 (IBM Corp., Armonk, NY, USA).

## Results

### Characterization of BM-MSCs

**Fig. 1:**
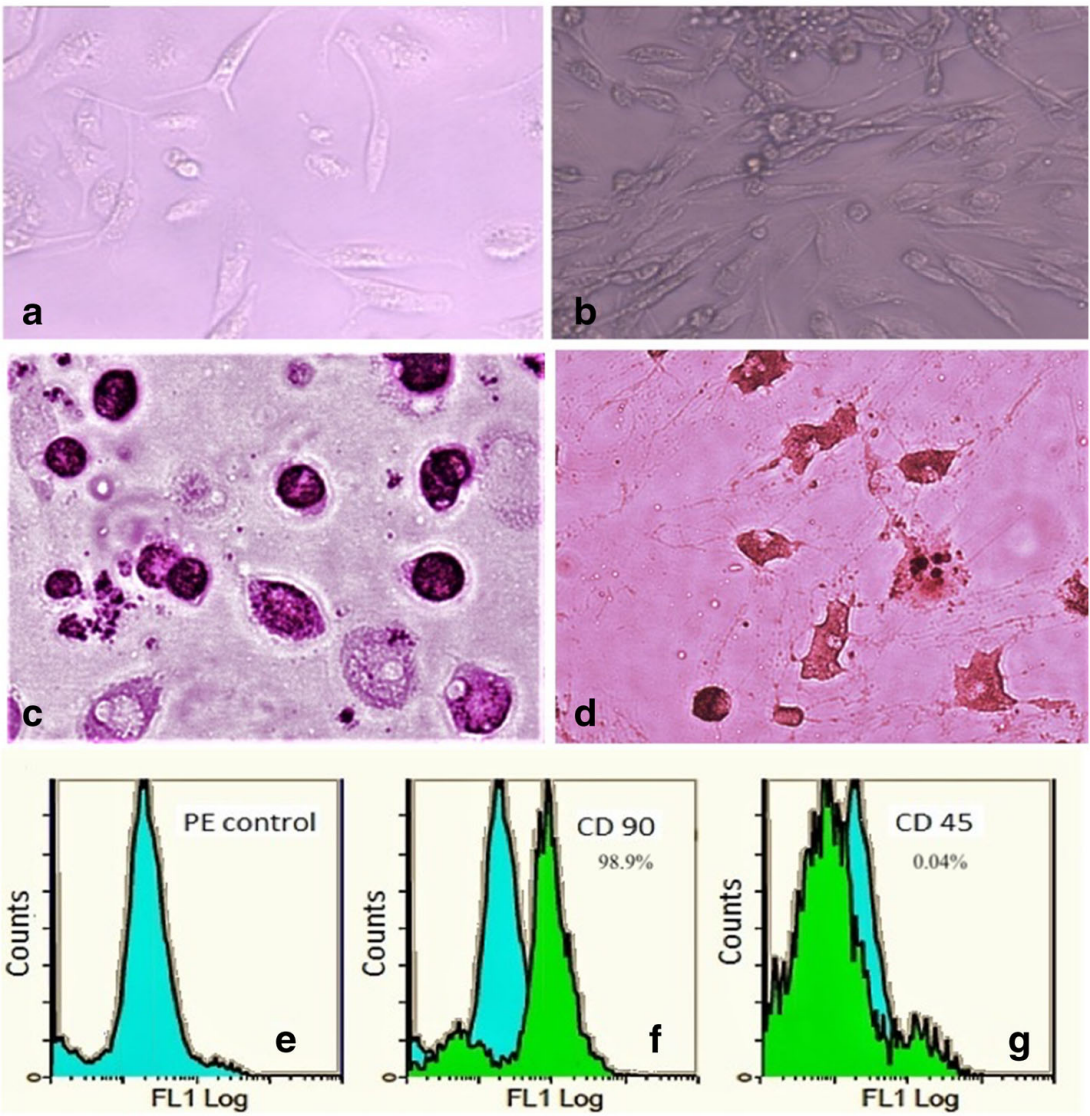
Bone marrow-derived mesenchymal stem cells (BM-derived MSCs) were characterized by morphology: BM-MSCs at 7 days of culture (**a**); BM-MSCs at 14 days of culture (**b**); BM-MSCs differentiated into osteocytes (**c**) and into adipocytes (**d**); flow cytometric characterization analyses of BM- MSCs: PE isotypic control (**e**); cells were uniformly positive for CD90^+^ and strongly expressed (**f**); cells were uniformly negative for CD 45^−^ and weakly expressed (**g**)

Cultured BM-MSCs were characterized as illustrated in Fig. 1 by having fusiform fibroblast-like cells: BM-MSCs at 7 days of culture (20–30% confluent) (Fig. 1a); BM-derived MSCs at 14 days of culture (80–90% confluent) (Fig. 1b); BM-MSCs differentiation into: osteocytes stained with Alizarin Red S (Fig 1c) and adipocytes stained with Oil Red O (Fig. 1d); and fluorescence-activated cell sorting (FACS) by assessment of PE isotypic control (Fig. 1e), positivity of CD90^+^ and strongly expressed (98.9%) (Fig 1f), and negativity of CD45^−^ and weakly expressed (0.04%) (Fig 1g) specific to MSCs.

### Effect on testicular weights and testosterone

Table 1 illustrated a significant weight loss in testis of the cisplatin group when compared with control rats. BM-MSCs injection significantly restored the testicular weight (*P* < 0.05). A significant decrease in plasma testosterone levels was observed in rats of cisplatin group when compared with normal group. BM-MSCs group significantly elevated the plasma testosterone levels in comparison to cisplatin group (*P* < 0.05).

**Table 1:** Effect of cisplatin and its combination with bone-marrow derived mesenchymal stem cells (BM-MSCs) on the weight of testis and testosterone levels. Data were presented as mean ± SD

Group	Normal	Cisplatin	BM-MSCs
Testis weight (g)	3.52 ± 0.5	2.75^*^ ± 0.23	3.38^#^ ± 0.15
Testosterone Levels (ng/dL)	120 ± 10.04	49^*^ ± 4.07	104^#^ ± 8.05

^*^Significant difference from the normal group at *p* < 0.05. ^#^Significant difference from cisplatin group at *p* < 0.05

### Effect on cisplatin-induced oxidative stress markers

Table 2 showed a significant elevation in testicular MDA (by 142.7%) and depletion in both GSH (by 42.1%) and SOD (by 56.9%) levels in rats injected with cisplatin alone in comparison to normal rats. However, rats injected with BM-MSCs showed attenuation in oxidative stress levels by a significant decrement in MDA testicular levels (by 53.1%) with a significant elevation of GSH (by 55.5%) as well as SOD (by 100.7%) testicular contents when compared with rats injected with cisplatin.

**Table 2:** Effect of cisplatin and its combination with bone-marrow derived mesenchymal stem cells (BM-MSCs) on testicular levels of malondialdehyde (MDA), reduced glutathione (GSH), and superoxide dismutase (SOD). All results were expressed as mean ± SD

Group	Normal	Cisplatin	BM-MSCs
MDA nmol/g tissue	36.55 ± 3.15	88.7^*^ ± 6.74	41.58^#^ ± 1.97
GSH mg/g tissue	94.29 ± 3.11	54.55^*^ ± 2.44	84.86^#^ ± 4.6
SOD U/g tissue	70.67 ± 3.4	30.44^*^ ± 2.3	61.1^#^ ± 2.9

^*^Significant difference from the normal group at *p* < 0.05. ^#^Significant difference from cisplatin group at *p* < 0.05

### Effect on inflammatory markers

To examine the effect of BM-MSCs on inflammatory markers, Table 3 determined the TNF-α protein content and iNOS gene expression in the testicular tissue of the studied groups. Cisplatin-treated rats showed a significant elevation in TNF-α testicular content (by 1.5-folds) in addition to upregulation of iNOS testicular expression (by 5.2-fold) when compared with normal control rats. The BM-MSCs injection showed a significant reduction in testicular TNF-α levels (by 40%) as well as downregulation of testicular iNOS expression (by 64.5%) when compared with cisplatin-treated rats.

**Table 3:** Effect of cisplatin and its combination with bone-marrow derived mesenchymal stem cells (BM-MSCs) on testicular protein levels of tumor necrosis factor alpha (TNF-α) and mRNA expression of inducible nitric oxide synthase (iNOS). All results were expressed as mean ± SD

Group	Normal	Cisplatin	BM-MSCs
TNF-α pg/mg protein	30.9 ± 5.1	77.11^*^ ± 10.1	46.21^#*^ ± 6.3
iNOS relative expression	0.1 ± 0.003	0.62^*^ ± 0.15	0.22^#^ ± 0.05

^*^Significant difference from the normal group at *p* < 0.05. ^#^Significant difference from cisplatin group at *p* < 0.05

### Effect on apoptotic markers gene expressions

**Fig. 2:**
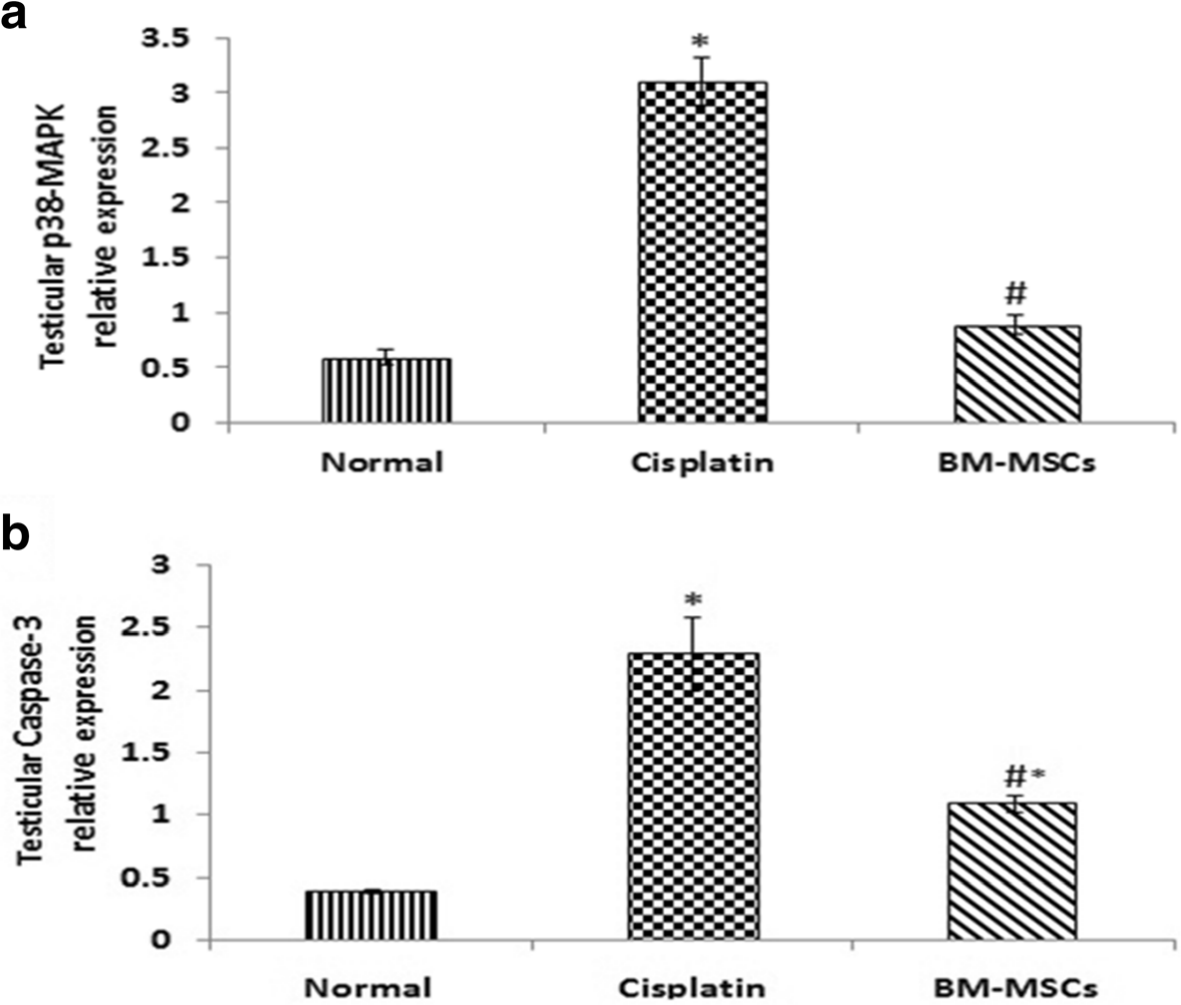
Effect of cisplatin and its combination with bone-marrow derived mesenchymal stem cells (BM-MSCs) on testicular mRNA expressions of p38- mitogen-activated protein kinase (p38-MAPK) (**a**) and caspase-3 (**b**). Data were presented as mean ± SD. ^*^Significant difference from the normal group at *p* < 0.05. ^#^Significant difference from cisplatin group at *p* < 0.05

To assess the role of apoptosis in our study we measured the expressions of apoptotic markers as illustrated in Fig. 2: p38-MAPK (Fig 2a) and caspase-3 (Fig. 2b) in testicular tissue and monitored the effect of BM-MSCs. Cisplatin-treated rats showed a marked upregulation in testicular expressions of p38-MAPK (by 4.3-fold) in addition to caspase-3 (by 4.7-fold) when compared with normal control rats. Rats treated with BM-MSCs showed a significant reduction in the expressions of testicular p38-MAPK (by 71.3%) and caspase-3 (by 52.2%) when compared with cisplatin-treated rats.

### Effect on seminiferous tubular diameters and histopathology

#### Seminiferous tubular diameter evaluation

**Fig. 3:**
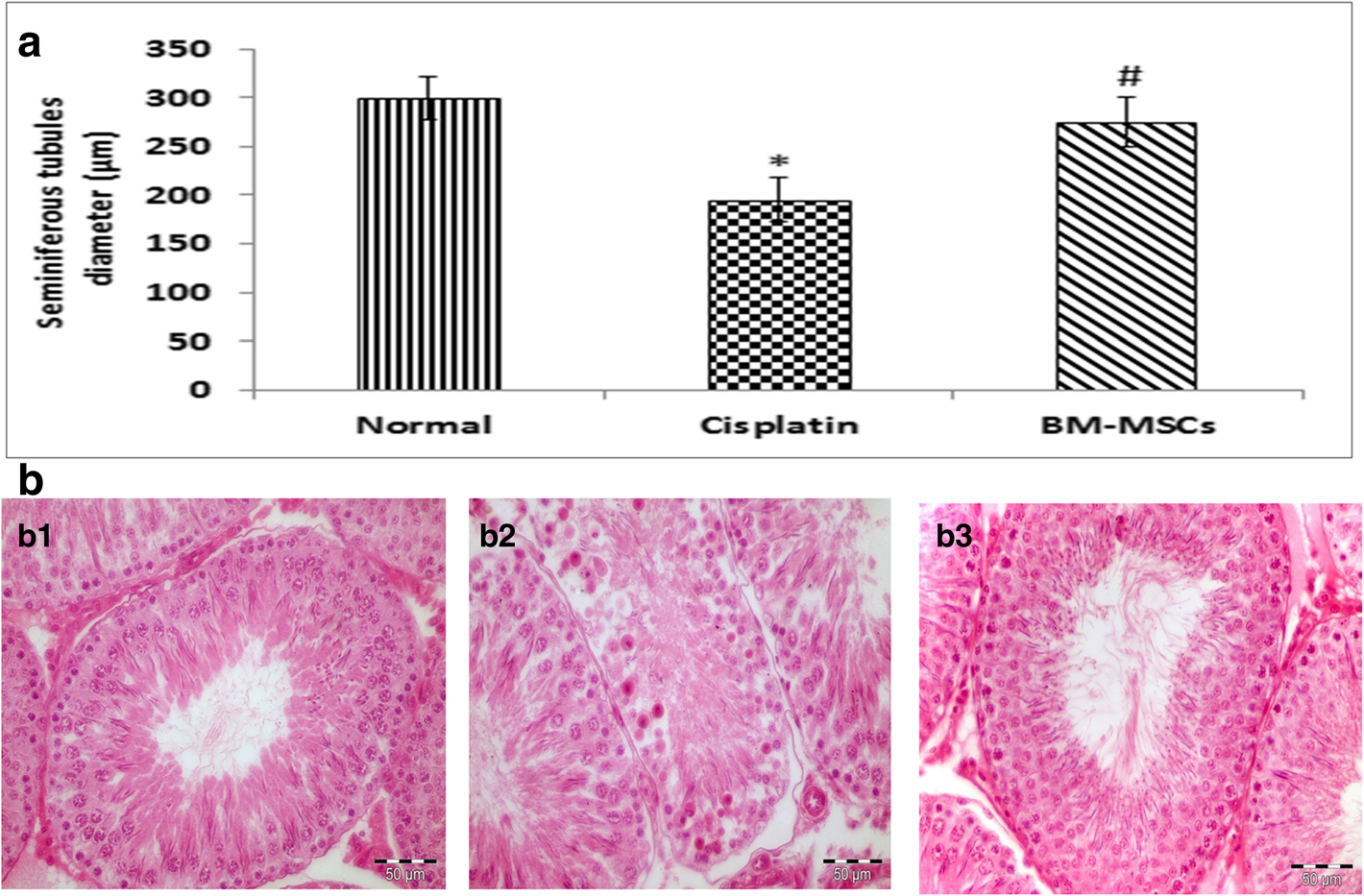
**a** Effect of cisplatin and its combination with bone-marrow derived mesenchymal stem cells (BM-MSCs) on mean seminiferous tubule diameter. Data were presented as mean ± SD ^*^Significant difference from the normal group at *p* < 0.05. ^#^Significant difference from cisplatin group at *p* < 0.05. **b** Cross-sections of the testes with hematoxylin and eosin staining (H&E ×400) showing regular seminiferous tubules (295 μm in diameter) with normal spermatogenesis, Sertoli cells, and mature spermatids in the normal group (**b1**); irregular seminiferous tubules (187 μm in diameter) with Sertoli cells only and maturation arrest. No evidence of spermatids in the cisplatin group (**b2**); regular seminiferous tubules (269 μm in diameter) with Sertoli cells, more germ cell layers, more spermatids near to that of normal in the stem cell group (**b3**)

The diameters of seminiferous tubules after cisplatin injection were decreased significantly in comparison to control normal rats (*P* < 0.05). A significant increase in seminiferous tubular diameters was observed in the BM-MSCs group in comparison to the cisplatin group (Fig. 3A).

#### Histomorphological findings

In Fig. 3B, normal control rats showed the normal architecture of the testes with regular seminiferous tubules and normal spermatogenesis, Sertoli cells and mature spermatids (Fig. 3B1). While rats treated with cisplatin were characterized by irregular small seminiferous tubules with Sertoli cells only and depletion of germ cells (Fig. 3B2). Rats in the stem cell group preserved nearly the spermatogenesis and showed regular seminiferous tubules of average diameters with Sertoli cells and more germ cells layers and more spermatids near to that of normal (Fig. 3B3).

### Effect on immunohistochemical staining of Bax expression

**Fig. 4:**
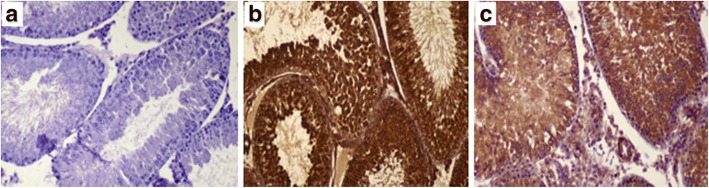
Effect of BM-MSCs injection on immunohistochemical staining of Bcl-2 associated x protein (Bax) (× 200). The Bax expression not detected in normal control group (**a**); marked expression in cisplatin group (**b**); and mild to moderate expression in BM-MSCs group (**c**)

On immunohistochemical evaluation (Fig. 4), brown staining in the cytoplasm of cells undergoing apoptosis in the mitochondrial region indicated positive Bax protein expression. The Bax expression could not be detected in control group (Fig.4a); marked Bax expression was observed in cisplatin-treated rats (Fig. 4b), and; mild to moderate Bax expression in BM-MSCs group (Fig. 4c).

## Discussion

The therapeutic potential of MSCs is based on its anti-inflammatory, antifibrotic, regenerative, and reparative properties; all could improve the damaged tissues [28]. Versatile therapeutic uses of MSCs were reported in the treatment and improvement of various diseases including wound healing, neurological and lung diseases, diabetes, cystic fibrosis, asthma, and infertility [16, 28]. In spite of their potential therapeutic applications, their use is associated with some concerns including sharing the characteristic of self-renewal and plasticity with cancer cells that may lead to tumor development [29]. Challenges such as durability of treatment and tumorigenesis mandate further studies to improve their therapeutic efficacy and applicability in clinical practice [30].

This work was done to show the possible beneficial role of BM-derived MSCs in cisplatin-induced testicular toxicity in rats. Our results proved that BM-derived MSCs ameliorated the toxicity of cisplatin on both molecular and pathological levels in testicular tissues. Several experimental studies have shown the beneficial effect of MSCs in testicular injuries induced by different agents [7, 12, 31–33]. This effect is mostly exerted via direct replacement of the damaged tissues and cell differentiation, or indirectly induces cell regeneration through paracrine signaling, which involves secretion of various mediators and growth factors that modulate the cellular interactions [16, 20, 34, 35].

In our study, there was a significant decrease in testicular weight and serum testosterone levels after cisplatin injection. Previous studies reported that cisplatin has a harmful effect on the testis and its gonadotoxicity is evident by testicular weight loss and reduced testosterone level [3, 5, 21, 35, 36]. The histopathological examination of our study explained the reduction in testis weight by reporting a moderate to severe gonadal atrophy with germ cells degeneration in the seminiferous tubules and a drastic reduction in tubular diameter after cisplatin injection. This result was in harmony with other studies [3–5, 21] confirming the testicular damage induced by cisplatin.

The injected MSCs improved both testicular weight and testosterone levels approaching normal levels. In animal models of testicular toxicity induced by busulfan and lead, BM-MSCs were used as a therapeutic potential and increased testosterone levels were observed [7, 32]. This increase might be due to differentiation of MSCs into steroidogenic cells as Leydig cells that produce testosterone [7, 31, 32, 37].

We proved in our previous study that cisplatin-induced testicular toxicity involves testicular oxidative stress, inflammation, and apoptosis [3]. In addition, many investigators documented the essential role of oxidative stress in the progression of testicular toxicity induced by cisplatin [3, 5, 36, 38, 39].

In the current study, markers for oxidative stress in testicular tissue were evaluated and we found that cisplatin administration caused a significant rise in MDA together with a remarkable reduction in GSH and SOD. This finding was inconsistent with other studies, which reported that cisplatin exposure induced disruption of the antioxidant system and ROS overproduction and this lead to depletion of cellular antioxidant defenses like reduced GSH and enzymes such as SOD and catalase in addition to increased lipid peroxidation and its product MDA [3, 40–42].

After MSCs injection, the disruption in previous oxidative stress markers was reversed. These findings coincide with other reports in which the antioxidative activities of MSCs were demonstrated [7, 43, 44].

Furthermore, it was reported that reactive nitrogen species is also involved in cisplatin-mediated gonadotoxicity [3]. Inducible nitric oxide synthase (iNOS) is accountable for the production of nitric oxide (NO) production that in turn initiates testicular toxicity [45]. The NO decreases intracellular GSH levels, which are considered one of the cellular defense mechanisms against toxic compounds and oxidative stress. Increased levels of iNOS were documented in our cisplatin-treated rats as documented by others [3, 36, 39, 46]. The BM-MSCs exhibited a significant downregulation in iNOS testicular expression reaching normal values thus counteracts the harmful side effect of cisplatin on rat testis, and this action may be attributed to the direct scavenging action of BM-MSCs on NO.

Oxidative stress played a major role in stimulating the inflammatory cascades including transcription factor nuclear factor kappa-B (NF-κB), which is considered as a junction between oxidative stress and inflammation [47, 48]. Production of inflammatory mediators like TNF-α and iNOS causes cytotoxic effects and trigger apoptosis and this intensifies the gonadotoxicity and testicular dysfunction induced by cisplatin [3, 42].

A significant increase of TNF-α testicular content was shown in cisplatin group when compared with normal control. A similar finding was noticed in previous studies [3, 39, 40, 42, 46], confirming the involvement of inflammation in reproductive toxicity induced by cisplatin.

Our experiment showed that BM-MSCs significantly lowered the TNF-α protein level when compared with cisplatin group. Similarly, we reported before a marked decline of renal TNF-α in rats treated with BM-MSCs after cisplatin injection [17]. This anti-inflammatory effect of MSCs is attributed to secretion of multiple soluble factors which have well known anti-inflammatory properties [34, 49].

On the other hand, the p38-MAPK pathway is known to be activated in response to various stresses involving oxidative stress and also it plays a key role in the development of reproductive toxicity induced by cisplatin [39]. Our results showed a significant upregulation in p38-MAPK testicular expression after cisplatin injection and this in agreement with other studies [3, 36, 46].

Meanwhile, apoptosis of the seminiferous tubular cells has participated in toxic gonadal damage. Caspases are family of enzymes involved in apoptosis. One of the caspases is caspase-3, which is considered the execution caspase and its activation lead to the initiation of the apoptotic cascades [22]. The Bax protein is a pro-apoptotic factor and is activated in conditions of oxidative stress-induced apoptosis [50]. In our study, significant upregulations in both caspase-3 mRNA expression and Bax protein expression were reported after cisplatin injection. Similar findings reported by Fouad and his colleagues in 2017 [42] showing a marked increase in caspase-3 protein levels and Bax/Bcl-2 ratio in rat testis treated with 10 mg/kg cisplatin, in addition, our research team reported before marked expressions of caspase-3 and Bax in rat kidney treated with 6 mg/kg cisplatin [17].

Our investigation showed that BM-MSCs are capable of reducing p38-MAPK, caspase-3, and Bax testicular expressions when compared with the cisplatin group. A recent study of Wang and his investigators in 2017 [14] suggested that the BM-MSCs repair effects on testicular toxicity induced by cadmium might be due to the inhibition of the apoptotic cascades including Bax/Bcl-2, Cytochrome C, and caspase-3 expressions. Furthermore, BM-MSCs showed a marked reduction in p38-MAPK, caspase-3, and Bax expressions in rat renal tissue treated with cisplatin [17, 18]. Downregulation of caspase-3 expression was also observed after BM-MSCs treatment of gentamicin-induced acute kidney injury [11, 48].

Hassan and Alam in 2014 proved that MSCs protect testicular tissue apoptotic damage in lead testicular toxicity [7]. They attributed this protective effect to the modulation of oxidative stress, tissue damage, and repair. These results supported that the transplanted MSCs have the ability to cause the release of various factors with antioxidative, antiapoptotic, anti-inflammatory and mitogenic activities [9, 49].

Finally, we still need to assess the benefit of repeated MSCs injection if repeated chemotherapy treatments are used. We believe that the MSCs injection could be combined with each timed injection, or it can be used at the end of chemotherapy cycles [51, 52]; however, future studies may answer this issue. In addition, the immune response against repeated MSCs injection is of great concern [53]. While MSCs are generally not immunogenic they clearly stimulate an immune response if given repeatedly in an environment with the right cytokines, especially if they reach the same inflammatory site.

## Conclusions

**Fig. 5:**
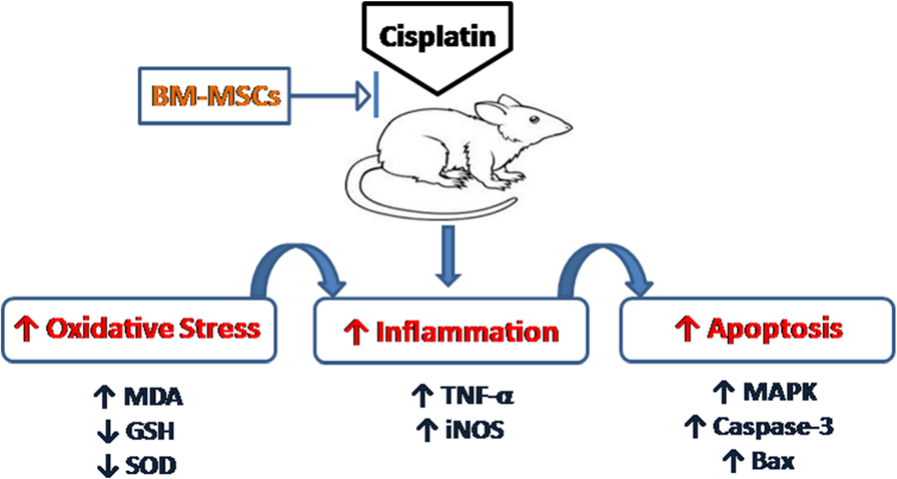
A schematic diagram showing the effect of BM-derived MSCs in platinum-induced gonadal damage. *Bax* Bcl-2 associated X protein, *BM-MSC* bone marrow-derived mesenchymal stem cells, *GSH* reduced glutathione, *iNOS* inducible nitric oxide synthase, *MDA* malondialdehyde, *p38-MAPK* p38-mitogen-activated protein kinase, *SOD* superoxide dismutase, *TNF-α* tumor necrosis factor alpha

The BM-MSCs could repair the gonadotoxicity induced by cisplatin through suppression of oxidative stress and inhibition of inflammatory and apoptotic cascades as summarized in Fig. 5. However, further studies should be conducted to validate our findings and to follow up the differentiation of BM-MSCs to evaluate their potency and durability at different time intervals.

## Abbreviations

Bax: Bcl-2 associated X protein
BM-MSCs: Bone marrow-derived mesenchymal stem cells
FACS: Fluorescence-activated analysis cell sorting
GSH: Reduced glutathione
IgG: Immunoglobulin G
iNOS: Inducible nitric oxide synthase
ip: Intraperitoneal
MAPK: Mitogen-activated protein kinase
MDA: Malondialdehyde
MSC: Mesenchymal stem cells
PBS: Phosphate-buffered saline
ROS: Reactive oxygen species
SOD: Superoxide dismutase
TNF-α: Tumor necrosis factor alpha
